# Recognizing Objects in 3D Point Clouds with Multi-Scale Local Features

**DOI:** 10.3390/s141224156

**Published:** 2014-12-15

**Authors:** Min Lu, Yulan Guo, Jun Zhang, Yanxin Ma, Yinjie Lei

**Affiliations:** 1 College of Electronic Science and Engineering, National University of Defense Technology, Changsha, Hunan 410073, China; E-Mails: lumin@nudt.edu.cn (M.L.); zhj64068@sina.com (J.Z.); newmyxin@163.com (Y.M.); 2 Department of Geography and Environmental Management, University of Waterloo, Waterloo, ON N2L 3G1, Canada; 3 College of Electronics and Information Engineering, Sichuan University, Chengdu 610064, China; E-Mail: leolyj@gmail.com

**Keywords:** object recognition, point cloud, local feature, clutter, occlusion

## Abstract

Recognizing 3D objects from point clouds in the presence of significant clutter and occlusion is a highly challenging task. In this paper, we present a coarse-to-fine 3D object recognition algorithm. During the phase of offline training, each model is represented with a set of multi-scale local surface features. During the phase of online recognition, a set of keypoints are first detected from each scene. The local surfaces around these keypoints are further encoded with multi-scale feature descriptors. These scene features are then matched against all model features to generate recognition hypotheses, which include model hypotheses and pose hypotheses. Finally, these hypotheses are verified to produce recognition results. The proposed algorithm was tested on two standard datasets, with rigorous comparisons to the state-of-the-art algorithms. Experimental results show that our algorithm was fully automatic and highly effective. It was also very robust to occlusion and clutter. It achieved the best recognition performance on all of these datasets, showing its superiority compared to existing algorithms.

## 1. Introduction

Object recognition is an active research topic in the area of computer vision [1,2]. It has a number of applications, including robotics, forensics, surveillance and remote sensing [3–5]. With the rapid development of 3D point cloud acquisition techniques, point clouds have became increasingly popular and available [6–9]. The aim of 3D object recognition is to correctly identify objects in a point cloud and estimate their 3D pose (*i.e.*, location and orientation) [10,11]. Although many algorithms have been proposed in the area of 3D object recognition, it is still very challenging to recognize objects in complex scenes in the presence of clutter and occlusion [10–12].

The existing 3D object recognition techniques can broadly be classified into global and local feature-based algorithms [4,11]. Global feature-based algorithms describe the whole surface of an object by a single descriptor. They require the scene point cloud to be pre-processed by a suitable 3D segmentation algorithm for the purpose of extracting individual object instances in the presence of clutter and/or occlusions [13]. They are frequently investigated in the area of shape classification and model retrieval [14]. In contrast, local feature-based algorithms have attracted more interests due to their robustness to clutter and occlusion [11,15,16]. Specifically, they first identify a number of keypoints in a scene and then extract a feature descriptor for each keypoint. These feature descriptors of the scene are finally matched against these feature descriptors of 3D models to get the recognition results.

Many existing local feature-based 3D object recognition algorithms use a set of single-scale features to represent a point cloud [10,17–21]. However, choosing an appropriate scale for a keypoint is very difficult. A large-scale feature contains sufficient information of the local surface of a keypoint at the cost of its high sensitivity to occlusion and clutter. On the contrary, a small-scale feature is very robust to occlusion and clutter. It however suffers from low descriptiveness. Scientific evidence from both physics and biological vision shows that multi-scale features are much more desirable with many attractive properties [22]. In this paper, we propose a multi-scale feature representation algorithm, which encodes an object by a set of local surface features with multiple scales. Experimental results show that our algorithm improves the 3D object recognition performance by a large margin compared to the state-of-the-art.

Once the scene and models are represented by local features, feature correspondences are established by matching scene features against model features. Three matching techniques have been proposed in the literature: threshold based, nearest neighbor (NN)-based and nearest neighbor distance ratio (NNDR)-based techniques [11,23,24]. In the case of threshold-based matching, two features are matched if the distance between their descriptors is less than a threshold. In the case of NN-based matching, two features are matched if the model feature descriptor is the nearest neighbor to the scene feature descriptor and if the distance between them is less than a threshold. In the case of NNDR matching, two features are matched if the model feature descriptor is the nearest neighbor to the scene feature descriptor and if the distance ratio between the first and second nearest neighbor is less than a threshold. The NNDR-based matching technique outperforms the other two techniques in terms of matching precision due to the fact that it penalizes the descriptors, which have many similar matches [23]. The NNDR-based matching technique has been widely used in image mosaic, 3D modeling, object recognition and 3D mapping [24,25]. One common limitation of all of these matching techniques is that an appropriate threshold should be determined before hand. The threshold is usually tuned by training experiments and is, therefore, dependent on the training data. This paper proposes a coarse-to-fine matching technique, which does not rely on selecting any specific threshold.

In this paper, we present an effective algorithm to recognize 3D objects in point clouds using multi-scale local surface features. Specifically, the contributions of the paper are as follows.

(i) We present a multi-scale feature representation algorithm to encode each scene/model. It first detects a number of keypoints in each scene/model and then extracts several feature descriptors with different scales at each keypoint. The proposed multi-scale feature representation is able to capture both the fine and coarse structures of a local surface.
(ii) We introduce a coarse-to-fine feature matching technique to establish feature correspondences between an input scene and models. It first uses a low threshold to produce a small number of accurate feature correspondences. It then increases the threshold to boost the number of feature correspondences. The proposed technique maintains a high level accuracy of feature matching while increasing the number of feature correspondences.
(iii) We develop a 3D object recognition framework based on the multi-scale feature representation and coarse-to-fine feature matching algorithms. The proposed method was tested on two publicly available datasets. Experimental results show that our method achieved high recognition rates. It was robust to clutter and occlusion and outperformed the state-of-the-art methods.

The rest of this paper is organized as follows. Section 2 describes the multi-scale feature representation algorithm. Section 3 introduces the coarse-to-fine feature matching algorithm and the 3D object recognition framework. Section 4 presents the experimental results for 3D object recognition in cluttered scenes with a comparison to existing techniques. Section 5 concludes the paper.

## 2. Multi-Scale Feature Representation

In this section, we present a multi-scale algorithm for object representation. The algorithm consists of two modules, *i.e.*, keypoint detection and feature description.

### 2.1. Keypoint Detection

**Figure 1.**
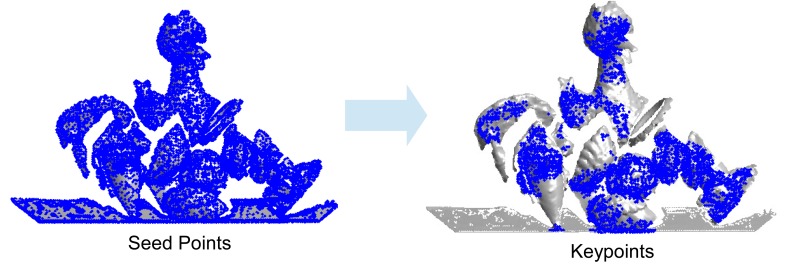
An illustration of the keypoint detection process.

The task of keypoint detection is to identify a set of interest points, which are distinctive and repeatable under a number of variations, including viewpoint changes, sensor noise, occlusion, clutter and point density variation [26]. In this paper, we detect keypoints based on our previous work [4]. Given a point cloud **P**, it is first converted into a triangular mesh **M**. The mesh is then decimated to obtain a low resolution mesh **Mˆ**. In this paper, we used the MATLAB function ‘reducepatch’ to reduce the number of faces of the original mesh while attempting to preserve the overall shape of the object. For all vertices of the decimated mesh **Mˆ**, their nearest points in the original mesh **M** are selected as seed points. The seed points are further pruned by a resolution control strategy [21] and a boundary checking algorithm [4,27]. In order to further improve the repeatability of keypoints, seed points with symmetric local surfaces are removed. The remaining seed points are finally considered keypoints. The whole process of keypoint detection is illustrated in Figure 1. It is clear that most of the keypoints are detected in the areas with large surface variation. Moreover, no keypoint can be found on the planar surface. This is important since the local planar surface contains very poor geometric information and is, therefore, not discriminative enough for feature description and object recognition.

### 2.2. Feature Description

**Figure 2.**
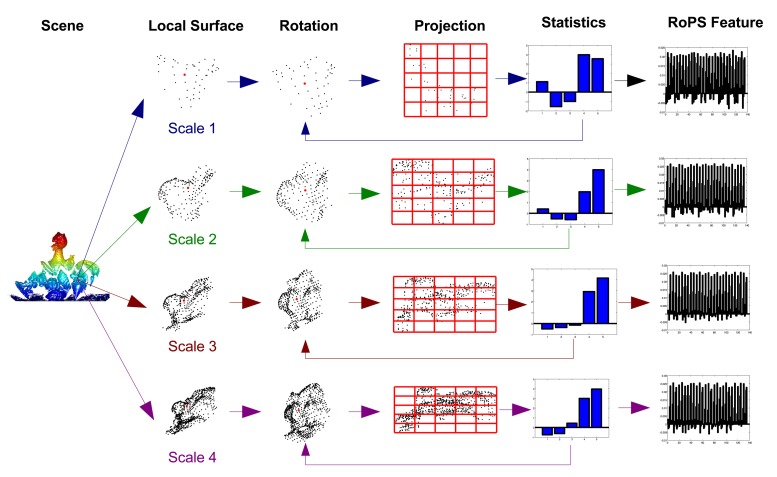
An illustration of the multi-scale rotational projection statistics (RoPS) feature description process (figure best seen in color).

Once a set of keypoints have been detected from a point cloud **P**, the next step is to describe the neighborhood of each keypoint with a feature descriptor. The descriptor projects the local surface around the keypoint into a proper feature space [26]. Descriptiveness and robustness are two critical qualifications for a local feature descriptor [11]. A number of feature descriptors have been proposed in the literature, including spin image [20], point's fingerprint [28], 3D shape context (3DSC) [29], snapshot [30], variable-dimensional local shape descriptors (VD-LSD) [19], Mesh Histogram of Oriented Gradients (MeshHOG) [31], exponential map (EM) [32] and rotational projection statistics (RoPS) [4,18]. For a comprehensive survey on local feature descriptors, the reader should refer to [11]. As reported in [4,11,33], RoPS achieves superior performance for feature matching in terms of precision and recall. It is also very robust to a set of variations, including Gaussian noise, shot noise, varying mesh resolutions and holes [4]. Therefore, we choose the RoPS algorithm for feature description in this paper. Different from our previous work [4,33] where fixed-scale RoPS features are used for 3D object recognition, this paper proposes a multi-scale RoPS feature representation. The process for generating multi-scale RoPS descriptors for a keypoint is illustrated in Figure 2.

Given a keypoint *p*, multiple scales {*r*_1_,*r*_2_, ⋯ , *r_n_s__*} are used to define the support radii of the keypoint. In our previous work, it is shown that the feature descriptors with a single scale of 15-times mesh resolution (mr) present the best overall performance. In order to encode more information around a keypoint, we use four different scales around 15 mr to achieve multi-scale representation in this paper (see more details in Section 3.5). For a selected scale *r_k_*, a local surface 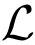
*_k_* is cropped from the mesh by using a sphere of radius *r_k_* centered at the keypoint ***p***. Assume that the local surface 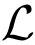
*_k_* consists of *n_t_* triangles and *n_v_* vertices and each triangle 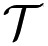
*_i_* contains vertices ***p****_i_*_1_, ***p****_i_*_2_ and ***p****_i_*_3_, the scatter matrix C*_i_* of each triangular face is calculated using all of the points lying within the triangle [4].

(1) $$\begin{array}{c} {\text{C}}_{i}=\frac{\int_{0}^{1}\int_{0}^{1-v}\left({\text{p}}_{c}\left(v,u\right)-\text{p}\right){\left({\text{p}}_{c}\left(v,u\right)-\text{p}\right)}^{\text{T}}\text{dudu}}{\int_{0}^{1}\int_{0}^{1-s}\text{dtds}}\\ =\frac{1}{12}\sum_{j=1}^{3}\sum_{k=1}^{3}\left({\text{p}}_{ij}-\text{p}\right){\left({\text{p}}_{ik}-\text{p}\right)}^{\text{T}}+\frac{1}{12}\sum_{j=1}^{3}\left({\text{p}}_{ij}-\text{p}\right){\left({\text{p}}_{ik}-\text{p}\right)}^{\text{T}} \end{array},$$

where:

(2) $${\text{p}}_{c}\left(v,u\right)={\text{p}}_{i1}+v\left({\text{p}}_{i2}-{\text{p}}_{i1}\right)+u\left({\text{p}}_{i3}-{\text{p}}_{i1}\right)$$

The scatter matrices of the *n_t_* triangles are then summed into an overall scatter matrix C. Next, an eigenvalue decomposition is applied to the overall scatter matrix to result in three eigenvectors:

(3) CV=EV

here, the diagonal entries of the matrix **E** correspond to the eigenvalues {λ_1_, λ_2_,λ_3_} of the scatter matrix **C**, and the matrix **V** consists of the three orthogonal eigenvectors {***v***_1_, ***v***_2_, ***v***_3_} of the scatter matrix **C**.

Finally, a sign disambiguation technique is performed on the three eigenvectors {***v***_1_, ***v***_2_, ***v***_3_}, resulting in three orthogonal and unambiguous vectors. That is, each unambiguous vector points in the direction of the scatter vectors [4]. These vectors are used to form a unique and repeatable local reference frame (LRF) for the local surface 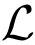
*_k_*.

Once the LRF for keypoint ***p*** with scale *r_k_* is generated, the local surface 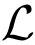
*_k_* is aligned with the LRF to achieve its invariance with respect to rigid transformations (*i.e.*, rotations and translations), resulting in a transformed local surface ${\text{L}}_{k}^{\prime }$. That is:

(4) $${\text{L}}_{k}^{\prime }={\text{R}}_{\text{lrf}}\left({\text{L}}_{k}-\text{p}\right)$$

where **R***_lrf_* is the rotation matrix defined by the LRF at the keypoint ***p***.

In order to encode the complete information of the local surface from different viewpoints, these points on ${\text{L}}_{k}^{\prime }$ are rotated along the three coordinate axes (*i.e.*, the *x-, y*- and *z*-axes). Along each axis, the points are rotated by a set of angles {*θ*_1_*, θ*_2_, ⋯, *θ_n_θ__*}, resulting in a resulted surface ${\text{R}}_{x}\left(\theta \right){\text{L}}_{k}^{\prime }$. The rotation matrix **R***_x_*(*θ*) along the *x*-axis is defined as:

(5) $${\text{R}}_{x}\left(\theta \right)=\left[\begin{array}{ccc} 1 & 0 & 0\\ 0 & \cos\left(\theta \right) & -\sin\left(\theta \right)\\ 0 & \sin\left(\theta \right) & \cos\left(\theta \right) \end{array}\right]$$

The rotation matrix **R***_y_*(*θ*) along the is the *y*-axis is defined as:

(6) $${\text{R}}_{y}\left(\theta \right)=\left[\begin{array}{ccc} \cos\left(\theta \right) & 0 & -\sin\left(\theta \right)\\ 0 & 1 & 0\\ \sin\left(\theta \right) & 0 & \cos\left(\theta \right) \end{array}\right]$$

The rotation matrix **R***_z_*(*θ*) along the is the *z*-axis is defined as:

(7) $${\text{R}}_{z}\left(\theta \right)=\left[\begin{array}{ccc} \cos\left(\theta \right) & -\sin\left(\theta \right) & 0\\ \sin\left(\theta \right) & \cos\left(\theta \right) & 0\\ 0 & 0 & 1 \end{array}\right]$$

Each rotation angle *θ* is defined between zero and 90 degrees. There is a tradeoff between the completeness and redundancy of the descriptor when selecting an appropriate number of rotation angles. Specifically, the descriptor with a larger number of rations encodes more information of the local surface. However, the information redundancy represented in the descriptor is also significantly higher. In our work, three rotations are used along each coordinate axis to achieve optimal overall performance.

For each rotation, these points are projected onto the three coordinate planes (*i.e.*, the *xy, yz* and *xz* planes). The projection process is defined as a mapping from a 3D space to a 2D space *ψ* : ℝ^3^ → ℝ^2^. A distribution matrix **D** is then obtained on each plane by counting the number of points falling into the bins of a *L* × *L* lattice. The value of *L* determines both the descriptiveness and the robustness of the extracted descriptor. That is, a smaller value of *L* encodes more detail of the local surface with higher sensitivity to varying mesh resolutions. In our previous work [4], it is demonstrated that *L* = 5 provides the best overall performance. Consequently, *L* = 5 is used in this paper. The distribution matrix **D** can further be encoded with few low-dimensional statistics. Different combinations of several statistics have been investigated in [4]; it is shown that the combination of five statistics (including four central moments [34] and one Shannon entropy [35]) achieves the best experimental performance. These statistics are invariant to rotations and translations.

The moment *μ_mn_* with the order *m* + *n* is calculated as:

(8) $$\mu_{mn}=\sum_{u=1}^{L}\sum_{v=1}^{L}{\left(u-\bar{u}\right)}^{m}{\left(v-\bar{v}\right)}^{n}\text{D}\left(u,v\right)$$

The entropy *e* is defined as:

(9) $$e=-\sum_{u=1}^{L}\sum_{v=1}^{L}\text{D}\left(u,v\right)\log\left(\text{D}\left(u,v\right)\right)$$

The total statistics of the distribution matrices on all planes (*i.e.*, the *xy, yz* and *xz* planes) with all rotations (*i.e., θ*_1_*, θ*_2_, ⋯, *θ_n_θ__*) are finally concatenated to form an overall RoPS feature descriptor. In order to represent the point cloud with multi-scale features, *n_s_* feature descriptors {***f***_1_, ***f***_2_,⋯, ***f****_n_s__*} are generated for each keypoint. That is, the feature descriptor *f_k_* is generated for keypoint *p* with scale *r_k_*.

## 3. Object Recognition

In this section, we propose a novel 3D object recognition framework based on multi-scale feature representation and coarse-to-fine feature matching techniques.

### 3.1. Object Recognition Framework

**Figure 3.**
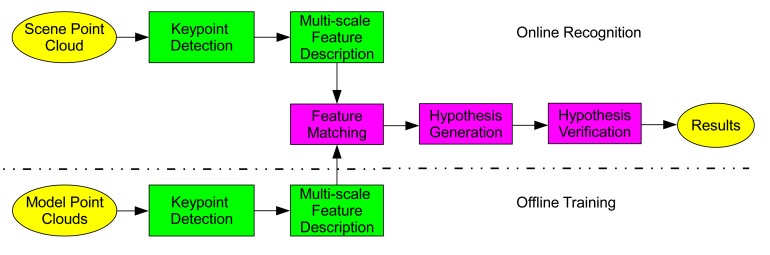
The pipeline of the 3D object recognition algorithm (figure best seen in color).

**Figure 4.**
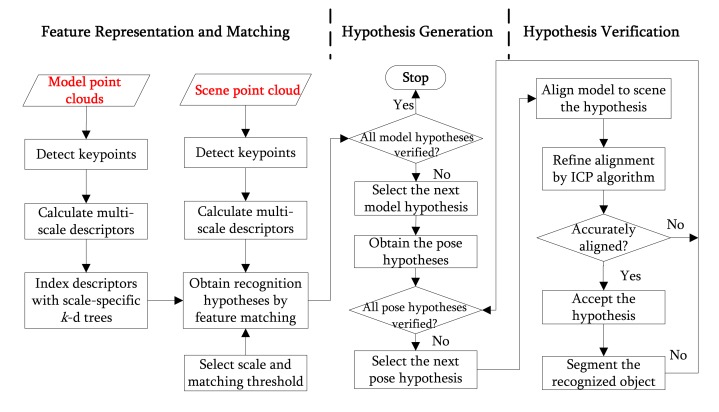
The flowchart of the 3D object recognition algorithm.

The pipeline of the 3D object recognition algorithm is shown in Figure 3, which consists of two major phases: offline training and online recognition. The flowchart of the 3D object recognition algorithm is presented in Figure 4.

During the phase of offline training, a model library 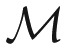 = { 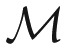
_1_, 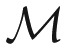
_2_, ⋯ , 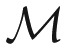
*_N_m__*}, which contains *N_m_* models for the 3D objects of interest, are constructed. For each model, 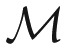
*_i_, n_m_* keypoints are first detected by uniform sampling and then pruned by a resolution control strategy [4,21]. For each keypoint ${\text{p}}_{m}^{i}$, its LRF ${\text{F}}_{mk}^{i}$ and RoPS descriptor ${\text{f}}_{mk}^{i}$ for each scale *r_k_* are calculated. In order to enable efficient feature matching during online recognition, *n_s_* scale-specific *k*-d trees are separately constructed to index the RoPS descriptors of all models. That is, for each scale *r_k_*, all RoPS descriptors that correspond to the scale *r_k_* are indexed with a *k*-d tree.

During the phase of online recognition, *n_p_* keypoints are detected from the scene 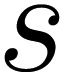 using the technique presented in Section 2.1. For each keypoint ***p****_s_*, its LRF **F***_sk_* and RoPS descriptor ***f****_sk_* for each scale *r_k_* are generated. Consequently, the scene is represented by a set of multi-scale RoPS descriptors {***f****_sk_*} (*s* = 1, 2, ⋯ , *n_p_.k* = 1, 2, ⋯ , *n_s_*), where *n_p_* is the number of scene keypoints and *n_s_* is the number of scales for each keypoint. We then propose a multi-scale feature matching strategy to produce a set of recognition hypotheses 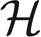 = {*h*_1_,*h*_2_, ⋯ , *h_n_h__*}. Each hypothesis *h_l_* is defined by a pair ( 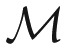
*_h_l__*, 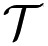
*_h_l__*), where 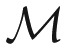
*_h_l__* is the model hypothesis and 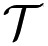
*_h_l__* is the pose hypothesis, which is used to transform 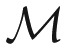
*_h_l__* to *S*. Given the hypotheses 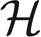, a hypothesis verification module is used to distinguish true hypotheses from false hypotheses, which further improves the rate of true positives while reducing the rate of false positives.

### 3.2. Feature Matching

We assume that the scene features are {***f****_sk_*} (*s* = 1, 2, ⋯ , *n_p_, k* = 1, 2, ⋯ , *n_s_*) and the model features are $\left\{{\text{f}}_{mk}^{i}\right\}$ (*i* = 1, 2, ⋯ , *N_m_, m* = 1, 2, ⋯ , *n_m_, k* = 1, 2, ⋯ , *n_s_*), where *n_p_* is the number of scene keypoints, *N_m_* is the number of models, *n_m_* is the number of model keypoints and *n_s_* is the number of scales for each keypoint. For each scene feature ***f****_sk_* with scale *r_k_*, it is matched against all model features in the library that have the same scale. Here, feature matching is performed using the previously-constructed *k*-d tree in order to speed up the process.

A scene feature ***f****_sk_* and a model feature ${\text{f}}_{mk}^{i}$ are matched if ${\text{f}}_{mk}^{i}$ is the nearest neighbor to ***f****_sk_* and if the distance ratio between the first and the second nearest neighbors is below a threshold *τ_f_*. In this paper, several values have been used for the threshold *τ_f_* to perform coarse-to-fine feature matching and object recognition (see Section 3.5 for more details). The scene feature ***f****_sk_* and its matched model feature ${\text{f}}_{mk}^{i}$ are considered a feature correspondence $\left({\text{f}}_{sk},{\text{f}}_{mk}^{i}\right)$. Each feature correspondence $\left({\text{f}}_{sk},{\text{f}}_{mk}^{i}\right)$ gives a vote to the *i*-th model. Then, the transformation (*i.e.*, pose estimation) ${\text{T}}_{\text{smk}}^{i}$ between the *i*-th model and the scene is calculated. The pose estimation ${\text{T}}_{\text{smk}}^{i}$ consists of a rotation matrix ${\text{R}}_{\text{smk}}^{i}$ and a translation vector ${\text{t}}_{\text{smk}}^{i}$, that is:

(10) $${\text{R}}_{\text{smk}}^{i}={\left({\text{F}}_{sk}\right)}^{\text{T}}{\text{F}}_{mk}^{i}$$

(11) $${\text{t}}_{\text{smk}}^{i}={\text{p}}_{s}-{\text{R}}_{\text{smk}}^{i}{\text{p}}_{m}^{i}$$

where ***p****_s_* is the scene keypoint, ${\text{p}}_{m}^{i}$ is the keypoint of the *i*-th model, **F***_sk_* is the LRF at the scene keypoint ***p****_s_* with the scale *r_k_* and ${\text{F}}_{mk}^{i}$ is the LRF at the model keypoint ${\text{p}}_{m}^{i}$with the scale *r_k_*.

### 3.3. Hypothesis Generation

For a given scale *r_k_* and matching threshold *τ_f_*, a set of feature correspondences can be generated. The models that have received votes from the feature correspondences are considered model hypotheses. For each model hypothesis 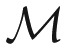
*_h_l__* , its associated pose estimations are then grouped into several clusters using the technique proposed in [4]. We calculate the cluster center (**R***_c_*, ***t****_c_*) for each cluster as the average value of all rotations and translations which fall in that cluster. Each cluster center is considered a pose hypothesis for the model hypothesis 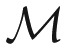
*_h_l__*. Note that, more than one cluster (*i.e.*, pose hypotheses 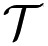
*_h_l__*) can be generated for each model hypothesis 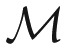
*_h_l__*.

### 3.4. Hypothesis Verification

Given the scene 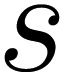 and hypotheses 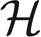 = {*h*_1_,*h*_2_,⋯*,h_n_h__*}, each hypothesis *h_l_* = ( 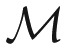
*_h_l__*, 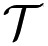
*_h_l__*) is verified as follows. First, the model 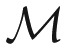
*_h_l__* is aligned with the scene 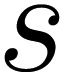 using the pose hypothesis 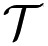
*_h_l__*. The alignment is further refined with an iterative closest point (ICP) algorithm [36]. The residual error *ε* of the ICP process is selected as a measure for the alignment. In addition, we define a visible proportion *α* as another measure, that is:

(12) $$\alpha =\frac{n_{\text{closest}}}{n_{\text{scene}}}$$

where *n_closest_* is the number of closest point pairs between 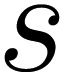 and 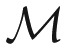
*_hl_* and *n_scene_* is the number of points in the scene 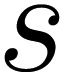.

The two measures *ε* and *α* are used to determine whether the hypothesis can be accepted or not. Ideally, for an object that is not occluded and its pose is accurately estimated, the residual error *ε* is zero and the visible proportion *α* is one. In practice, two thresholds *τ_ε_* and *τ_α_* are used to determine an acceptable hypothesis. In order to accept as many correct hypotheses as possible while reducing false positives, a flexible thresholding scheme is used in this paper to perform hypothesis verification. Specifically, two groups of thresholds (*τ*_*ε*1_ = 0.75 mr, *τ*_*α*1_ = 0.04) and (*τ*_*ε*_2 = 1.5 mr, *τ*_α_2 = 0.2) are adopted, where ‘mr' denotes the average mesh resolution. These thresholds were determined by a tuning experiment, and the same values were applied to all experiments presented in the paper. The hypothesis ( 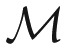
*_h_l__*, 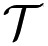
*_h_l__*) is accepted only if *ε < τ*_*ε*1_ and *α > τ*_*α*1_ or *ε < τ*_*ε*2_ and *α > τ*_*α*2_. Otherwise, the hypothesis is rejected.

### 3.5. Coarse-to-Fine Recognition

Most existing algorithms generate feature descriptors using a single scale and perform feature matching using a pre-defined threshold *τ_f_*. They however have a number of limitations. First, it is difficult to choose an appropriate scale for a fixed-scale feature-based 3D object recognition algorithm. That is, feature descriptors with a large scale are very sensitive to occlusion and clutter (which is common in most object recognition scenarios). In contrast, feature descriptors with a small scale lack rich descriptiveness. Second, although many adaptive-scale keypoint detection algorithms have been proposed in the literature (e.g., Mesh Difference of Gaussians (MeshDoG) [31], keypoint quality-adaptive scale (KPQ-AS) [27] and salient points (SP) [37]), their scale repeatability is low [26]. For example, the scale repeatability of MeshHoG, KPQ-AS and SP algorithms is, respectively, 41%, 51% and 43%, when tested on the University of Western Australia (UWA) laser scanner dataset [26]. Consequently, the performance of object recognition is adversely affected by the errors of scale estimation. Third, the pre-defined threshold *τ_f_* is data-dependent and very difficult to determine. That is, although selecting a strict threshold can produce highly accurate feature correspondences, the number of feature correspondences may be too few to perform effective object recognition. On the contrary, selecting a loose threshold would produce lots of false feature correspondences, which not only increases the computational time, but also deteriorates the accuracy of object recognition.

**Figure 5.**
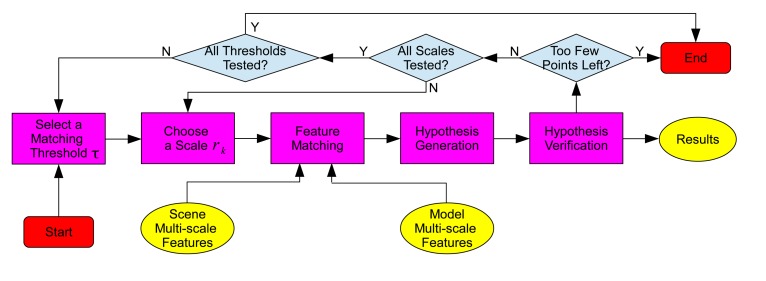
Coarse-to-fine object recognition algorithm (figure best seen in color).

In this paper, a coarse-to-fine algorithm is proposed to solve these problems. The algorithm is illustrated in Figure 5. Multiple scales (*i.e.*, 5 mr, 10 mr, 15 mr and 20 mr) and different matching thresholds *τ_f_* (*i.e.*, 0.7, 0.8, 0.9 and 1.0) are used in the algorithm. Here, ‘mr' stands for the average mesh resolution. Note that the values of the aforementioned thresholds were selected by a tuning experiment and were applied to the experiments on all datasets (Section 4). First, the algorithm uses large-scale features (with a scale of 20 mr) and a strict threshold (with a value of 0.7) to perform feature matching (Section 3.2), hypothesis generation (Section 3.3) and hypothesis verification (Section 3.4). If part of the hypotheses are accepted by the algorithm, the instances of these model hypotheses are recognized from the scene, and the scene points that belong to these model hypotheses are removed. Once all resulting hypotheses are verified, the object recognition algorithm then proceeds to features with a smaller scale, while keeping the matching threshold fixed. The aforementioned feature matching, hypothesis generation and hypothesis verification modules are then repeated. Once features of all scales for a fixed matching threshold are tested, the algorithm proceeds to a looser matching threshold. The aforementioned process continues until either too few points left in the scene for recognition or all scales and thresholds have been tested.

The strengths of the coarse-to-fine algorithm are as follows. First, the objects with large visible parts in the scene can be recognized with a high priority, since the object recognition algorithm starts with matching of large-scale features. Consequently, most of these highly visible objects can be segmented from the scene within a few iterations, which significantly reduces the overall recognition time. Second, the object with small visible parts in the scene can also be easily recognized due to the reason that most of the large objects have already been segmented in advance. That is, there are very few clutter points left in the scene. As a result, the recognition rate is increased. Third, the feature correspondences are sequentially verified by the order of their distinctiveness. That is, the most distinctive feature correspondences are verified with a strict threshold before these less distinctive ones. As a result, the computational efficiency is improved, and the number of correct feature correspondences is also increased.

## 4. Experimental Results

**Figure 6.**
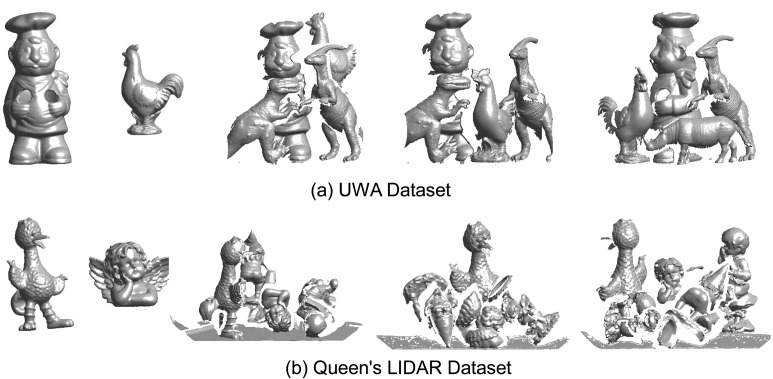
Example images of the University of Western Australia (UWA) and Queen's datasets.

The proposed algorithm is tested on two publicly available datasets, *i.e.*, the University of Western Australia (UWA) dataset [10] and the Queen's LiDAR dataset [19,38]. Some example images of the two datasets are shown in Figure 6.

### 4.1. Recognition Results on the UWA Dataset

The UWA dataset is currently regarded as the most popular benchmark for 3D object recognition [4,10,11,13,19,27,32]. It consists of five models and 50 scenes. Each scene contains four or five of the models in the presence of occlusion and clutter. Specifically, four or five models were first selected and randomly placed on a table, and a point cloud was then acquired by the triangulation based Konica Minolta Vivid 910 scanner from a single viewpoint. The total number of instances of each object in all scenes is shown in Table 1. The recognition rate of each object on the UWA dataset is also presented in Table 1. It can be observed that chef, chicken and T-rex achieved the highest recognition rate of 100%. Besides, Parasaurolophus and rhino also obtained a high recognition rate of more than 96%. Specifically, both of them had only one instance left in the scene that was not correctly recognized. The failure of these two instances was due to being highly occluded. The overall recognition rate of the five objects is 99.1%. The results clearly confirm that the proposed coarse-to-fine recognition algorithm is capable of recognizing objects in complex scenes in the presence of multiple objects, occlusion, clutter and noise.

**Figure 7.**
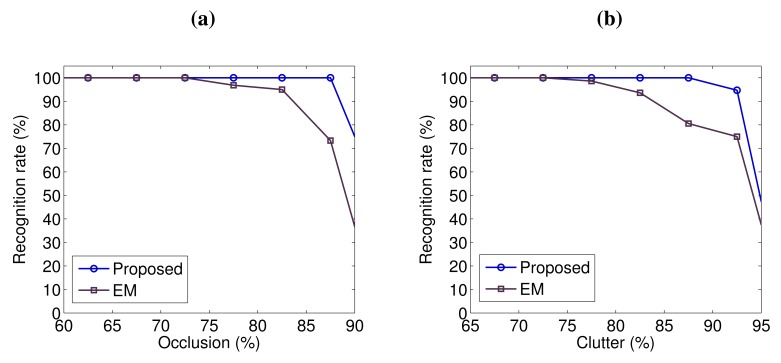
Recognition rates of the five objects on the 50 scenes of the UWA dataset. (**a**) Recognition rates with respect to occlusion; (**b**) Recognition rates with respect to clutter.

In order to further analyze the robustness of our algorithm with respect to occlusion and clutter, we present the recognition rates of the five objects on the 50 scenes in Figure 7, as a function of occlusion and clutter. The results reported by the EM-based algorithm [32] are also shown in Figure 7. According to the definitions presented in [10,32], occlusion is calculated as:

(13) $$\text{occlision}=1-\frac{\text{model surface patch area in scene}}{\text{total model surface area}}$$

Clutter is calculated as:

(14) $$\text{clutter}=1-\frac{\text{model surface patch area in scene}}{\text{total surface area of scene}}$$

We can observe that our algorithm is very robust to occlusion and clutter. It achieved a high recognition rate of 100% with up to 87.5% occlusion and 87.5% clutter. Its recognition rate was still as high as 94.7% with up to 92.5% clutter. Our algorithm clearly outperformed the EM-based algorithm [32], especially on the scenes with high values of occlusion and clutter. These comparative results further demonstrate the effectiveness of our algorithm for object recognition in the presence of significant occlusion and clutter.

**Figure 8.**
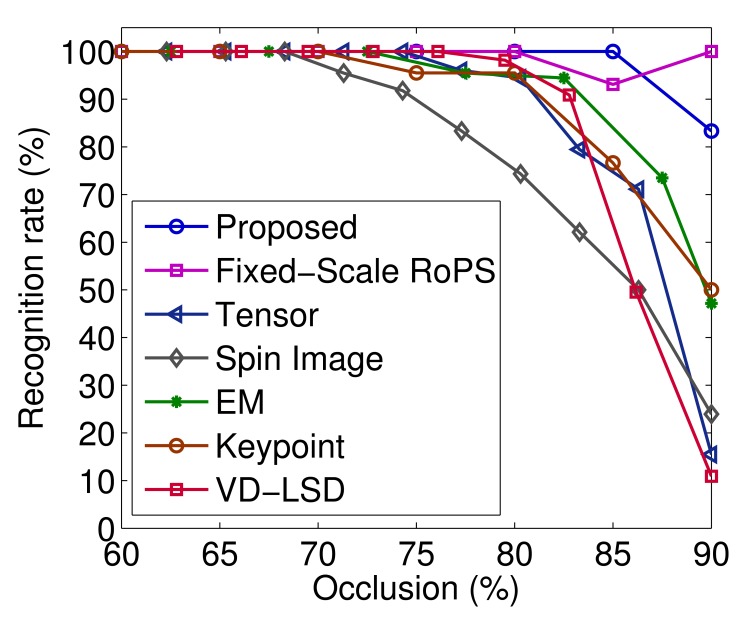
Recognition rates of the four objects on the 50 scenes of the UWA dataset with respect to occlusion (figure best seen in color).

In order to perform a rigorous and fair comparison with the state-of-the-art algorithms [4,10,32], we compare our results with the recognition results presented in [4,10,32] on exactly the same dataset of cluttered scenes. That is, we excluded the model rhino from our recognition results. That is because the spin image-based algorithm failed to recognize the rhino in any of these scenes (as discussed in [10]). Figure 8 shows the recognition rates of the remaining four objects with respect to occlusion. The results reported by tensor [10], spin image [10], keypoint [27], VD-LSD [19], EM [32] and fixed-scale RoPS [4] -based algorithms are also presented in Figure 8. The recognition rate of our algorithm in this case was 100% with up to 84% occlusion. In contrast, the recognition rates of tensor [10], spin image [10], EM [32] and fixed-scale RoPS [4] -based algorithms were respectively 96.6%, 87.7%, 97.5% and 98.8%, with up to 84% occlusion. The proposed algorithm obtained the best overall recognition rate of 99.5%, followed by the fixed-scale RoPS-based algorithm (with an overall recognition rate of 98.9%).

**Figure 9.**
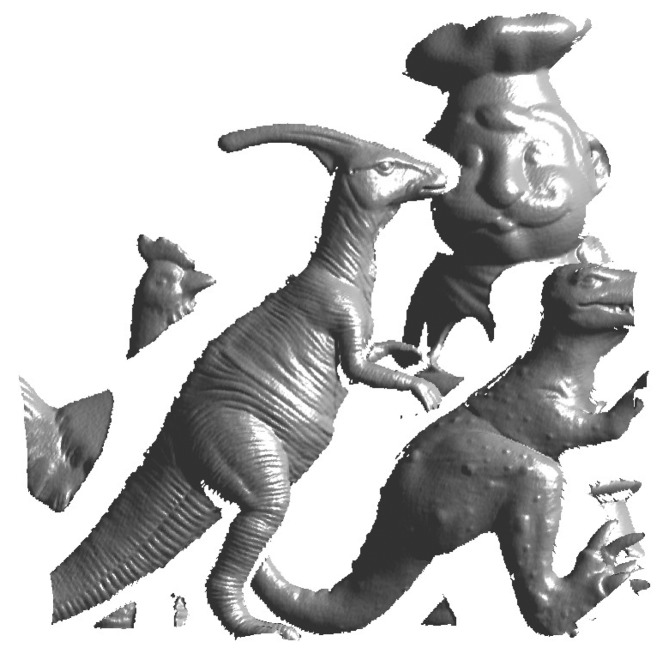
An illustration of the scene with occlusion of an object (*i.e.*, the chicken model) by more than 85%. EM, exponential map; VD-LSD, variable-dimensional local shape descriptors.

The superior performance of our algorithm is due to several facts. First, our RoPS feature outperforms the state-of-the-art local surface features in terms of recall and precision [4]. It is also very robust to a set of variations, including clutter, occlusion, noise and varying mesh resolutions, as demonstrated in [4]. Consequently, both fixed-scale and multi-scale RoPS feature-based algorithms achieved better performance compared to the others (as shown in Figure 8). Second, multi-scale RoPS features are capable of encoding both coarse and fine structures of an object. They are, therefore, more effective for the purpose of object recognition compared to their fixed-scale counterparts (as shown in Figure 8). Specifically, large-scale features are more suitable for the efficient recognition of objects with small occlusion. In contrast, small-scale features are more appropriate for the robust recognition of objects with large occlusion. An illustration of the scene with high occlusion (*i.e.*, the chicken model) is shown in Figure 9. Finally, our coarse-to-fine recognition algorithm uses multiple thresholds for feature matching rather than a single threshold. It therefore, produces more hypotheses and ultimately improves the recognition accuracy

### 4.2. Recognition Results on Queen's LIDAR Dataset

The Queen's LIDAR dataset is composed of five models and 80 scenes. Each scene was generated by placing one, three, four or five of the models in a scene and was scanned from a single viewpoint using a Konica-Minolta Vivid 3D scanner [19]. The objects in each scene are highly cluttered, where the clutter includes both other objects and background [19]. We first tested our algorithm on the full dataset, which contains 80 scenes. Table 2 shows our recognition rates of the five objects on the dataset, with a direct comparison to the results achieved by EM [32] and fixed-scale RoPS [4] -based algorithms. It is clear that our algorithm achieved a recognition rate of 100% for all objects in that dataset. The second best place is taken by the fixed-scale RoPS-based algorithm, with an average recognition rate of 95.4%. In contrast, the performance achieved by the EM-based algorithm is relatively low, with an average recognition rate of 82.4%. It can be inferred that the proposed multi-scale RoPS feature-based algorithm further improves the performance of 3D object recognition compared to the fixed-scale RoPS feature-based algorithm [4].

In order to have a fair comparison with the results reported by EM-, VD-LSD-, 3DSC-, spin image- and fixed-scale RoPS-based algorithms, we tested our coarse-to-fine 3D object recognition algorithm on the same dataset as [4,19,32]. The selected dataset is actually a subset of the full Queen's LiDAR dataset. The subset dataset contains only 55 scenes. Each scene consists of three, four or five objects. Our recognition rates of the five objects on this subset dataset are shown in Table 3. We also present the results reported by fixed-scale RoPS-, EM-, VD-LSD with scalar quantization (VD-LSD-(SQ)) , VD-LSD with vector quantization (VD-LSD-(VQ)) , 3DSC-, spin image- and spin image spherical-based algorithms in Table 3. Similar to the results achieved on the full dataset, our coarse-to-fine algorithm obtained a recognition rate of 100% for all objects on this subset dataset. It is better than the fixed-scale RoPS-based algorithm by a margin of 4.6% in the average recognition rate. This observation fully indicates that the proposed multi-scale RoPS-based coarse-to-fine algorithm outperforms the algorithm that uses fixed-scale RoPS features and a single matching threshold. As compared with other algorithms, the advantage of the proposed algorithm is even more significant. That is, the average recognition rates reported by all other algorithms (except fixed-scale RoPS) are less than 85%.

## 5. Conclusions

In this paper, we proposed a coarse-to-fine algorithm for 3D object recognition in point clouds. We used multi-scale RoPS features to represent an object and performed 3D object recognition based on feature matching, hypothesis generation and hypothesis verification. We employed several different matching thresholds to conduct feature matching in order to further improve the object recognition performance. The proposed algorithm was evaluated on two publicly available datasets. Experimental results show that our algorithm outperformed existing algorithms in terms of recognition rates. It is shown that the algorithm is also very robust to occlusion and clutter. In our future work, we aim to investigate the challenging task for the recognition of geometrically featureless objects/scenes (e.g., with planar surfaces). One of the prospective solutions is to integrate geometric and photometric information for object recognition.

## Figures and Tables

**Table 1.** Recognition rate of each object on the UWA dataset.

	**Chef**	**Chicken**	**Parasaurolophus**	**T-Rex**	**Rhino**	**Overall**
Number of Instances	50	48	45	45	28	216
Recognition Rate (%)	100	100	97.8	100	96.4	99.1

**Table 2.** Recognition rates on the full Queen's dataset. The best results are in bold face.

**Algorithm**	**Angel** (%)	**Big-Bird** (%)	**Gnome** (%)	**Kid** (%)	**Zoe** (%)	**Average** (%)
Proposed	**100**	**100**	**100**	**100**	**100**	**100**
Fixed-Scale RoPS [4]	97.9	100	97.7	95.8	85.4	95.4
EM [32]	77.1	87.5	87.5	83.3	76.6	82.4

**Table 3.** Recognition rates on a subset of the Queen's dataset. The best results are in bold face. 3DSC, 3D shape context.

**Algorithm**	**Angel** (%)	**Big-Bird** (%)	**Gnome** (%)	**Kid** (%)	**Zoe** (%)	**Average** (%)
Proposed	**100**	**100**	**100**	**100**	**100**	**100**
Fixed-Scale RoPS [4]	97.4	100	97.4	94.9	87.2	95.4
EM [32]	NA	NA	NA	NA	NA	81.9
VD-LSD (SQ) [19]	89.7	100.0	70.5	84.6	71.8	83.8
VD-LSD (VQ) [19]	56.4	97.4	69.2	51.3	64.1	67.7
3DSC [19]	53.8	84.6	61.5	53.8	56.4	62.1
Spin Image [19]	53.8	84.6	38.5	51.3	41.0	53.8
Spin Image Spherical [19]	53.8	74.4	38.5	61.5	43.6	54.4
